# Correction for Sulaiman et al., “The Brief Case: A fatal case of *Legionella pneumophila* in an elderly patient”

**DOI:** 10.1128/jcm.01847-24

**Published:** 2025-01-08

**Authors:** Andrew Sulaiman, Emily Gisriel, Karen C. Carroll

## AUTHOR CORRECTION

Volume 62, no. 11, e00863-24, 2024, https://doi.org/10.1128/jcm.00863-24. Page 7: In the answer to the first self-assessment question, we may have erroneously implied that currently marketed urinary antigen tests are FDA cleared for detection of serogroups 1, 3, 6, 8, and 12. While the ImmuView assay is approved only for detection of *L. pneumophila* serogroup 1, it may detect the other serogroups listed if present in high numbers.

